# Cost-Effectiveness of Adjuvanted Influenza Vaccine Compared with Standard and High-Dose Influenza Vaccines for Persons Aged ≥50 Years in Spain

**DOI:** 10.3390/vaccines13030323

**Published:** 2025-03-19

**Authors:** Alberto Perez-Rubio, Roberto Flores, Jesus Ruiz Aragon, Javier Sanchez, Sergio Marquez-Peláez, Piedad Alvarez, Andres Osorio Muriel, Joaquin Mould-Quevedo

**Affiliations:** 1 Hospital Clínico Universitario de Valladolid, 47003 Valladolid, Spain; albertoprz@gmail.com; 2 Medical Scientific Liaison, CSL Seqirus, 08027 Barcelona, Spain; roberto.flores@seqirus.com; 3 Hospital Universitario Puerta del Mar, 11009 Cádiz, Spain; reducido@hotmail.com; 4 Modeling and Simulation, Evidera, Torre Nozar, Titan, 15, 28045 Madrid, Spain; 5 Department of Economics, Economic Analysis, Faculty of Business Pablo de Olavide University, 41013 Seville, Spain; smarpel@upo.es; 6 Evidence, Modeling, and Synthesis, Evidera, 500 Totten Pond Road, Waltham, MA 02451, USA; piedad.alvarez@evidera.com; 7 Modeling and Simulation, Evidera, Bogota CO Calle 90, Bogota 110221, Colombia; andres.osoriomuriel@evidera.com; 8 Global Health Economics, CSL Seqirus, Summit, NJ 07901, USA

**Keywords:** influenza, vaccination, Spain, cost-effectiveness, adjuvanted, high-dose, burden of illness, age ≥ 50 years

## Abstract

Background: The prevalence of chronic conditions that increase the risk of influenza complications is high among individuals aged ≥50 years, and evidence suggests age-related changes in immune responses to vaccines begin to decline at this age. Persons aged 50–59 years have high rates of influenza infections and are also the most likely age group to be employed. Thus, the burden of influenza is high in this age group. Methods: We investigated the cost-effectiveness of vaccination with an adjuvanted quadrivalent influenza vaccine (aQIV) in a Spanish population aged ≥50 years at high risk of influenza complications. Using a static decision-tree model specifically designed to analyze Spanish data, we calculated incremental cost-effectiveness ratios (ICERs) for aQIV vs. egg-based QIV (QIVe; indicated for any age) and aQIV vs. high-dose QIV (HD-QIV; indicated for persons aged ≥60 years) from payer and societal perspectives. We compared ICERs against a willingness-to-pay threshold of EUR 25,000 per quality-adjusted life year (QALY) gained. The impact of input uncertainty on ICER was evaluated through a probabilistic sensitivity analysis (PSA) and a one-way deterministic sensitivity analysis (DSA). Results: The total incremental cost of vaccination with aQIV was EUR –86,591,967.67, which was associated with gains of 241.02 in QALY (EUR –359,268.05 per QALY gained) and 318.04 in life years (EUR −272,271.37 per life year gain). Compared with the willingness-to-pay threshold of EUR 25,000 per QALY gained, aQIV was the most cost-effective influenza vaccine relative to the combination of QIVe or HD-QIV. These findings were supported by PSA and DSA analyses. Conclusions: In the model, aQIV dominated QIVe and HD-QIV, demonstrating that aQIV use would be cost-saving for persons aged ≥50 years who are at high risk of influenza complications.

## 1. Introduction

Since the global impact of the coronavirus disease 2019 (COVID-19) pandemic has begun to wane, influenza has resumed its place as a leading cause of hospitalizations and deaths worldwide in the 2023–2024 influenza season [1,2]. Historically, seasonal influenza was associated with 13% of all respiratory disease deaths per year, in association with 1 billion influenza cases annually [3,4]. Similarly to other countries, the costs of influenza in Spain are high, amounting to EUR 1 billion per year in direct medical costs and indirect costs such as absences from work due to influenza illness or caring for family members with the disease [5].

Rates of hospitalizations and mortality are highest among those aged ≥65 years [6,7,8,9]. Persons aged ≥50 years have the highest rates of influenza infections [10]. Out of all age groups, those aged 50–59 years have the highest rate of employment in Spain [11], and thus absences due to their own or a family member’s illness can have a major impact on the overall burden of influenza [10,12]. The prevalence of chronic, cardiometabolic diseases, which increase the risk of influenza complications, is also high in adults aged ≥50 years [13,14,15,16], potentially contributing to increased influenza severity and the costs of medical care in this age group [12]. In addition, age-related changes in immune function that reduce immune responses to vaccination (known as immunosenescence) may begin as early as age 50 years [17,18].

Annual influenza vaccination is recommended in Spain for adults aged ≥60 years as well as younger persons at high risk of influenza complications due to chronic diseases and other medical conditions [19]. Currently, the population younger than 60 years of age may be given a standard-dose, non-adjuvanted egg-based influenza vaccine containing 15 μg of hemagglutinin from each of the influenza virus strains contained within the vaccine. Two vaccines specifically developed to overcome age-related decreases in immune response are authorized for use in older adults [19,20]. The adjuvanted influenza vaccine contains the MF59^®^ adjuvant in addition to the standard 15 μg dose of hemagglutinin for each influenza strain in the vaccine and is authorized for persons aged ≥65 years. In older adults, the MF59 adjuvant produces broader, more robust, and more durable immune responses compared with the standard influenza vaccine [21,22]. MF59-adjuvanted influenza vaccines have also been shown to provide greater immunogenicity against both homologous and heterologous influenza strains vs. standard vaccines in persons younger than 65 years [23]. In addition, a high-dose influenza vaccine, containing 60 μg of hemagglutinin for each selected influenza virus, is authorized for individuals aged ≥60 years [19]. In this population, the high-dose vaccine produces a stronger immune response relative to standard influenza vaccines [24].

In Spain, persons aged 50–59 years, including those at high risk of influenza complications, typically receive standard-dose influenza vaccine. Because of the excess burden of influenza in this population, we sought to investigate the cost-effectiveness of adjuvanted influenza vaccine in high-risk persons aged 50–59 as well as adults aged ≥60 years.

## 2. Materials and Methods

### 2.1. Model Design

The health economic model used in this study was based on a static decision-tree model designed for use in Spain according to Spanish best practices for health economic modeling [25,26]. Similar models have been used for influenza vaccine cost-effectiveness analyses in other countries [27,28,29,30,31], while previous studies have used the Spanish model to estimate health economic endpoints related to influenza vaccination in Spain among children and adults aged ≥9 years at risk of influenza complications and among older adults aged ≥65 years [26,32,33,34,35]. In this analysis, we used the model to simulate the costs and burden of influenza disease and the costs and benefits of influenza vaccination with the adjuvanted quadrivalent influenza vaccine (aQIV), the egg-based quadrivalent influenza vaccine (QIVe), or the high-dose quadrivalent influenza vaccine (HD-QIV) among residents of Spain aged ≥50 years during a single influenza season.

### 2.2. Model Inputs and Calculations

**Figure 1:**
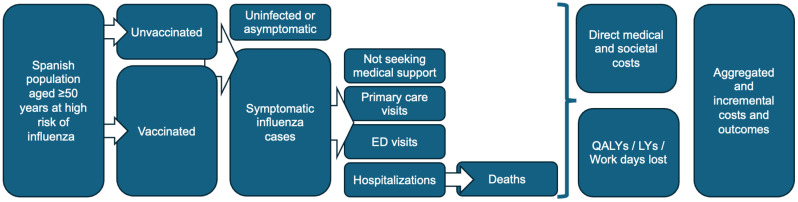
Schematic of the health economic model. ED, emergency department; LY, life years; QALY, quality-adjusted life year. Adapted from Ruiz-Aragón et al. *Vaccines (Basel)*. 2022; 10:176 [32].

Figure 1 displays a schematic of the model inputs. The Spanish population aged ≥50 years was divided into the following age groups: 50–59, 60–64, 65–74, and ≥75 years, which were further divided into subgroups based on high or low risk of influenza complications (Table 1). All individuals aged ≥60 years were included in the high-risk group.

The model analysis focused on the high-risk population and excluded persons aged 50–59 years at low risk of influenza complications. In the model, those aged ≥50 years were either vaccinated or unvaccinated. Based on vaccination coverage rates for each age group, vaccinated individuals were divided into two comparator arms, one where they received either a QIVe or an HD-QIV according to their age group indication (50–59 or ≥60 years, respectively) and another arm where all people aged ≥50 years received an aQIV. Over a one-year time horizon representing a single influenza season, the vaccinated and unvaccinated populations were divided into the following subgroups: uninfected or asymptomatic; symptomatic cases not seeking medical support; or symptomatic cases requiring either a primary care visit, emergency department visit, or hospitalization. Each subgroup was assigned a fixed cost and disutility associated value, and hospitalized patients were assigned a probability of death. The totals for each cohort for the entire influenza season were calculated by aggregating the outcomes and costs (direct medical and societal) across the different subgroups. All costs are expressed in 2024 euros. Following Spanish cost-effectiveness guidelines, productivity loss due to death and quality-adjusted life year (QALY) loss due to death were calculated over a lifetime horizon and discounted at 3% per year [25]. The incidence of influenza cases per 100,000 Spanish residents over seven influenza seasons (2013–2020) was based on data from the Instituto de Salud Carlos III Centro Nacional de Microbiología (ISCIII) (Table S1) [39,40,41,42,43,44,45]. Mortality data over the same time period appear in Table S2 [46]. Table 2 lists inputs used in the model, and Table S3 shows the population estimates on which employment-related figures in Table 2 are based. Table S4 provides the basis for the population of older adults requiring care by a family member. The tender price of QIVe used in the model was based on the weighted average of the prices in each region (Table S5).

The base case relative vaccine effectiveness (rVE) estimate for the aQIV vs. the HD-QIV was 1.40% (95% confidence interval [CI], −1.7% to 4.4%), based on a 2022 meta-analysis of head-to-head vaccine studies conducted in the ≥65 years of age population [78]. This rVE estimate is consistent with values determined in multiple real-world evidence studies, including test-negative design studies comparing the effectiveness of the aQIV vs. the HD-QIV for the prevention of laboratory-confirmed influenza cases [79,80,81,82,83,84,85,86]. We assumed the rVE was the same (1.40%) for the 60–64 year age group. The rVE used in the base case was in line with the first randomized study between the aQIV and the HD-QIV, which demonstrated that the two vaccines did not differ in effectiveness against laboratory-confirmed influenza [87]. For the aQIV vs. the QIVe, the base case rVE was 20.0% (95% CI, 2–34%) in the ≥65 year age group [88], and we assumed the same benefit would exist for those aged 50–59 years as no rVE estimates for the aQIV vs. the QIVe in persons younger than 64 years have yet been published.

### 2.3. Analysis

The model outputs include costs, burden of illness (symptomatic cases, primary care visits, emergency department [ED] visits, hospitalizations, and deaths in persons vaccinated with the aQIV or their age-indicated vaccine [QIVe or HD-QIV]), and incremental analysis. Costs comprised public health system costs, discounted societal costs, and total discounted QALYs. Public payer costs were not discounted because they were calculated over only one year, whereas societal costs and QALY losses due to death were calculated based on life expectancy and discounted accordingly. Incremental cost-effectiveness ratios (ICERs) for the aQIV vs. the QIVe or the HD-QIV were calculated from a direct medical payer and societal perspective.

ICERs were also compared against a willingness-to-pay threshold of EUR 25,000 per QALY gained, which is the cost-effectiveness range used by the National Health Service in Spain [89,90]. To assess the effect of uncertainty on the ICERs, a probabilistic sensitivity analysis (PSA) was conducted in which parameters were varied based on their CIs over 10,000 iterations of the model. A one-way deterministic sensitivity analysis (DSA) was also used to evaluate the impact of input uncertainty on ICER. Finally, a second sensitivity analysis was conducted using 13 alternative modeling scenarios involving different cost and benefit discount rates, the rates of influenza vaccine coverage among high- and low-risk groups, the types of influenza vaccines used in different age groups, and the costs of the QIVe (Table S6). In addition, the alternative scenarios included rVEs for the aQIV vs. the HD-QIV that utilized age group–specific estimates from the Domnich et al. meta-analysis (7.8% for ages 60–64 and 65–74 years and 12.5% for ≥75 years) [78] and lower (13.9%) [91] and higher values (34.6%) [92] for the rVE of the aQIV vs. the QIVe (Table S6).

## 3. Results

As shown in Table 3, 9,094,819 individuals at high risk of influenza complications were vaccinated with either the QIVe or the HD-QIV (according to age-based indications) in the current scenario or with the aQIV in the alternative scenario of the model. In the 50–59 year age group, aQIV vaccination cost EUR 9.6 million more than QIVe vaccination, but costs of the aQIV were lower than costs of HD-QIV vaccination in all groups aged ≥60 years.

In the cost-effectiveness model analysis (Table 4), the aQIV dominated vaccines as currently indicated, resulting in reduced QALY and life years lost; fewer influenza cases, influenza-related outpatient and inpatient medical visits, and deaths; and lower direct medical costs related to influenza (Table 4). The total incremental savings of vaccination with the aQIV was EUR 86,591,967.67, which was associated with gains of 241.02 in QALY (EUR −359,268.05 per QALY gained) and 318.04 in life years (EUR −272,271.37 per life year gain).

**Figure 2:**
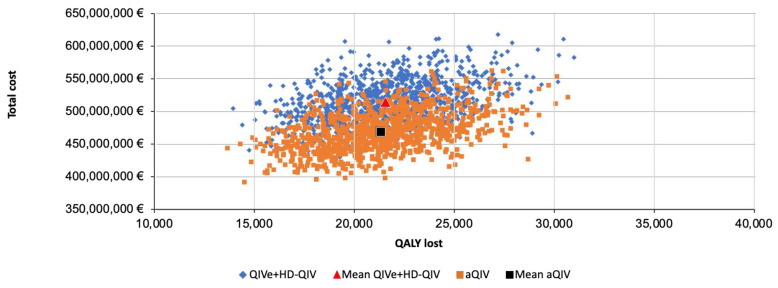
Results of the probabilistic sensitivity analysis (PSA) in persons at high risk of influenza complications. aQIV, adjuvanted quadrivalent influenza vaccine; HD-QIV, high-dose quadrivalent influenza vaccine; QALY, quality-adjusted life year; QIVe, egg-based quadrivalent influenza vaccine.

The PSA confirmed that costs and QALY lost were lower with the aQIV than with the QIVe plus the HD-QIV across the range of 95% CI values for model parameters (Figure 2).

**Figure 3:**
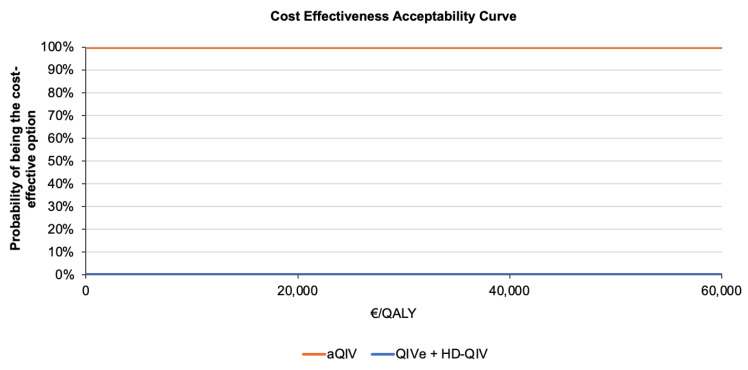
Cost-effectiveness acceptability curve comparing the probability of being the most cost-effective option between the aQIV (intervention) and control, i.e., either the QIVe given to persons aged 50–59 years who are at high risk of influenza complications or the HD-QIV administered to persons aged ≥60 years. aQIV, adjuvanted quadrivalent influenza vaccine; HD-QIV, high-dose quadrivalent influenza vaccine; QALY, quality-adjusted life year; QIVe, egg-based quadrivalent influenza vaccine.

When compared against the willingness-to-pay threshold of EUR 25,000 per QALY gained, aQIV had a 100% probability of being the most cost-effective influenza vaccine relative to the combination of QIVe or HD-QIV (Figure 3).

**Figure 4:**
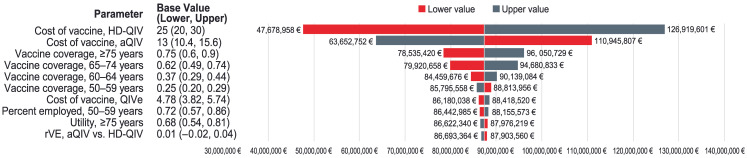
Tornado diagram showing the incremental net monetary benefit for the aQIV vs. the QIVe (age 50–59 years at high risk of influenza complications) or the HD-QIV (age ≥60 years) at a willingness-to-pay threshold of EUR 25,000 per QALY. aQIV, adjuvanted quadrivalent influenza vaccine; HD-QIV, high-dose quadrivalent influenza vaccine; QIVe, egg-based quadrivalent influenza vaccine; rVE, relative vaccine effectiveness.

In the DSA (Figure 4), vaccine costs were shown to have the greatest impact on ICER, followed by vaccine coverage in each high-risk age group, in descending order. Other parameters had relatively little effect on cost-effectiveness.

The results of the additional sensitivity analysis, testing the model with different inputs, generally supported the dominance of vaccination with the aQIV over vaccination with the QIVe or the HD-QIV in high-risk persons aged ≥50 years (Table S7). The aQIV was found to be more effective in all scenarios and dominant in terms of both cost and effectiveness in 10 of 13 scenarios (public health system perspective, discount rates of 0% and 1.5%, different vaccine coverage rates per Garcia et al. 2016 [75], QIVe and HD-QIV given to 50–59 and ≥75 year age groups, respectively, alternate rVE point estimates, and lower and higher prices for the QIVe).

## 4. Discussion

This modeling analysis is the first to estimate the cost-effectiveness of using the aQIV in persons aged ≥50 years in any country. The results support the use of the aQIV in persons aged 50–59 years, who would normally receive the QIVe, and those aged ≥60 years, who would receive the HD-QIV in Spain. Administration of the aQIV to the high-risk population reduced the incidence of symptomatic cases of influenza, inpatient and outpatient medical encounters, deaths, and all associated costs. These savings offset the higher tender price of the aQIV relative to the QIVe for the group aged 50–59 years and amplified the cost savings of the aQIV relative to the higher tender price for the HD-QIV for those aged ≥60 years.

In Spain, 72% of persons aged 50–64 years are employed [11]. Due to age and experience, many of these individuals may hold positions of seniority at their workplace, lending an outsized impact on their absence from work due to illness or the need to care for a family member. A systematic literature review has shown that influenza-associated indirect costs, including absenteeism due to either infection or caring for infected family members, are higher in the 50–64 year age group than in adults aged ≤50 years [12]. At the same time, the rates of chronic diseases such as obesity, diabetes, and cardiovascular disease, which increase the risk of influenza complications and death, begin to climb at age ~50 years, and half of middle-aged adults have at least two high-risk conditions [13,14,15,16]. In addition, immunosenescence begins to emerge after age 50 and will affect most individuals aged ≥60 years [17,18].

Despite the burden of influenza in the 50–64 year age group, many countries’ health authorities do not cover, or even recommend, influenza vaccination for persons younger than 60 or 65 years, particularly for healthy individuals. Yet multiple cost-effectiveness analyses conducted in both Northern and Southern Hemisphere nations support influenza vaccination for all persons 50–64 years, with the cost of vaccination falling well below cost-effectiveness thresholds from both payer and societal perspectives, even if the population is at low risk of influenza complications [93,94,95,96,97]. A recent analysis from the UK described the benefits of expanding vaccination with the cell-based quadrivalent influenza vaccine (QIVc) to low-risk persons aged 50–64 years. Using a dynamic transmission model calibrated to actual infection data from the UK, the authors showed that vaccinating this age group was cost-saving due to reductions in influenza disease costs [95]. Another modeling study demonstrated that vaccinating low-risk individuals between 50 and 64 years of age reduces burdens on the healthcare system, including bed usage in acute and intensive care wards [96].

Our modeling analysis focused on high-risk persons aged ≥50 years because the burdens of influenza are highest among these individuals. Previous modeling analyses conducted with the Spanish population aged ≥65 years have demonstrated the cost-effectiveness of strategies using adjuvanted influenza vaccines. One analysis showed that reductions in influenza-associated medical costs offset the price difference between the adjuvanted vaccine and the standard-dose vaccine [98], whereas another demonstrated that use of adjuvanted vaccines would lead to EUR 82 million in savings for Spanish health systems, with a cost–benefit ratio of 12.83 [99]. An analysis conducted in 2021 that compared the aQIV to the HD-QIV in persons aged ≥65 years demonstrated cost savings of EUR 64.2 million [32]. A dynamic model designed to account for herd immunity showed that the aQIV is more cost-effective than the QIVe in the ≥65 population [34]. In another recent analysis, the aQIV was likewise shown to be more cost-effective than the recombinant quadrivalent influenza vaccine (QIVr) [33]. Our findings are consistent with all of these studies and further demonstrate that these benefits are extended to high-risk persons aged 50–59 years. Another study published in 2021 that also analyzed the cost-utility between high-dose and adjuvanted vaccines concluded that HD-QIV use in persons aged ≥65 years was at least cost-effective, if not dominant, over the adjuvanted trivalent influenza vaccine (aTIV) in Spain [35]. However, this study has important limitations. In the absence of a clinical trial comparing the high-dose and adjuvanted vaccines, the authors used indirect comparisons from a clinical trial and meta-analysis of the HD-TIV vs. standard influenza vaccine over several seasons, along with a one-season observational study of the aTIV vs. a virosomal vaccine [24,100,101]. This indirect comparison resulted in an rVE that was not supported by a meta-analysis of head-to-head, real-world evidence (RWE) studies comparing the aTIV with the HD-TIV or standard influenza vaccines [78,91]. During the review of the current article, a new meta-analysis has been published that again concludes that no difference in benefit was observed between aQIV and HD-QIV [102]. Results from a recent randomized, head-to-head pragmatic trial of the HD-QIV vs. the aQIV with laboratory-confirmed influenza are consistent with the RWE meta-analysis [87].

When we conducted this analysis, no efficacy data from randomized controlled trials or effectiveness data from real-world studies were available for the aQIV in persons aged 50–64 years. Due to this limitation, we assumed that the rVE point estimates from studies in the ≥65-year-old population would be the same for the population aged 50–64 years. We also assumed hospitalization costs would be the same in all age groups. However, overall rates of influenza-related hospitalization are highest among adults aged ≥65 compared to all other age groups, whereas the costs of hospitalization for individuals aged 50–64 years are often higher on a per-case basis, possibly because middle-aged adults who require hospitalization for influenza have more severe complications [12]. Recently, the B/Yamagata influenza strain has disappeared from circulation [103]. Like some other health authorities, the Spanish Ministry of Health has recommended switching to trivalent formulations for influenza vaccines used in future seasons [19]. Our analysis focused on quadrivalent influenza vaccines, as recommended in recent seasons. It is unlikely the presence or absence of a fourth strain would have affected our results, as most prior studies have focused on trivalent vaccines. The change from quadrivalent to trivalent vaccines might, however, affect the analysis if the change to trivalent causes a price change, and the Spanish Medicines Agency must approve the new prices.

## 5. Conclusions

Our findings suggest that vaccinating the population aged ≥50 years against influenza in Spain will result in cost savings. In addition, we demonstrate the benefits of the aQIV in high-risk persons aged 50–59 years, who are normally eligible for the standard-dose QIVe. Vaccination with the aQIV vs. the QIVe reduces influenza disease burden, including symptomatic cases, outpatient medical visits, ED visits, hospitalizations, and deaths—as well as associated costs. These cost reductions more than offset the increased tender price of the aQIV relative to the QIV, supporting the cost-effectiveness of the aQIV for persons aged ≥50 years who are at high risk of influenza complications.

## Figures and Tables

**Table 1:** Characteristics of the population analyzed in the model [36,37,38].

Age Group	Total Population N	Life Expectancy Years	High Risk n (%)	Low Risk n (%)	Influenza Vaccine Coverage, High Risk %
50–59 years	7,475,397	30.6	4,770,798 (63.8)	2,704,599 (36.2)	24.54%
60–64 years	3,214,012	23.6	3,214,012 (100)	0	36.72%
65–74 years	4,974,175	17.9	4,974,175 (100)	0	61.57%
≥75 years	4,890,766	6.4	4,890,766 (100)	0	75.27%

**Table 2:** Model inputs.

Parameter		Value		Reference
aQIV tender price		EUR 13		[47]
QIVe tender price ^a^		EUR 4.78		[48,49,50,51,52,53,54,55,56,57,58,59,60,61,62,63,64,65,66,67]
HD-QIV tender price		EUR 25		[47]
Primary care physician visit costs (per visit)		EUR 65.00		[68]
ED visit costs (per visit)		EUR 257.00		[68]
Hospitalization costs (per event)		EUR 5809.61		[69]
Nurse consultation cost		EUR 25.94		[70]
No. business days of disability	Primary care physician visit	5		[71]
	ED visit	5		[71]
	Hospitalization	15 ^b^		[72]
Employment rate	0–50 years	65.8%		[11]
	50–59 years	71.6%		[11]
	60–64 years	48.9%		[11]
	65–74 years	10.0%		[11]
	≥75 years	0.8%		[11]
Proportion requiring care at home	65–74 years	18.4%		[73]
	≥75 years	45.1%		[73]
Proportion aged 18–64 who care for a family member and are also employed		35.15%		[73]
Productivity loss per day ^c^	0–50 years	EUR 106.58		[74]
	50–59 years	EUR 126.86		[74]
	60–64 years	EUR 129.78		[74]
	65–74 years	EUR 129.78		[74]
	≥75 years	EUR 129.78		[74]
Baseline utility		High risk	Low risk	
	0–50 years	0.940	0.975	[75]
	50–59 years	0.870	0.960	[75]
	60–64 years	0.870	0.960	[75]
	65–74 years	0.836	0.955	[75]
	≥75 years	0.676	0.836	[75]
Disutility value for symptomatic patients (all ages)		0.32		[76]
Disutility value for outpatient and ED settings		High risk	Low risk	
	0–50 years	0.42	0.40	[77]
	50–59 years	0.42	0.40	[77]
	60–64 years	0.46	0.44	[77]
	65–74 years	0.37	0.35	[77]
	≥75 years	0.33	—	[77]
Disutility value for an inpatient setting (all ages)		0.42	0.40	[77]
Disutility duration for symptomatic patients		7 days		[76]
Disutility duration in an outpatient setting		7 days		[77]
Disutility duration in an inpatient setting		7 days		[77]
Discount rate for costs and outcomes ^d^		3%		[25]

aQIV, adjuvanted quadrivalent influenza vaccine; ED, emergency department; HD-QIV, high-dose quadrivalent influenza vaccine; QALY, quality-adjusted life year; QIVe, egg-based, standard-dose influenza vaccine. ^a^ Weighted average of tender prices across Spanish regions. ^b^ Equivalent to the QALY loss for 3 weeks. ^c^ Productivity loss per day is calculated as the labor cost per hour (EUR 16.22) times daily labor hours (8 h). ^d^ Productivity loss due to death and quality-adjusted life year loss due to death are calculated over a lifetime horizon and discounted at 3% per year.

**Table 3:** Costs of influenza vaccines and vaccine administration in persons at high risk of influenza complications aged ≥50 years in Spain.

			Current Scenario ^a^		Alternative Scenario ^a^	
Costs	Age Group	Number Vaccinated	QIVe	HD-QIV	(aQIV)	Incremental Difference
Influenza vaccines	50–59 years	1,170,754	EUR 5,596,204		EUR 15,219,801	EUR 9,623,597
	60–64 years	1,180,185		EUR 29,504,630	EUR 15,342,408	EUR −14,162,222
	65–74 years	3,062,600		EUR 76,564,989	EUR 39,813,794	EUR −36,751,195
	≥75 years	3,681,280		EUR 92,031,989	EUR 47,856,634	EUR −44,175,355
	Total ^b^	9,094,819	EUR 203,697,812		EUR 118,232,637	EUR −85,465,175
Vaccine administration	50–59 years	1,170,754	EUR 30,369,357		EUR 30,369,357	EUR 0
	60–64 years	1,180,185		EUR 30,614,004	EUR 30,614,004	EUR 0
	65–74 years	3,062,600		EUR 79,443,832	EUR 79,443,832	EUR 0
	≥75 years	3,681,280		EUR 95,492,392	EUR 95,492,392	EUR 0
	Total ^b^	9,094,819	EUR 235,919,585		EUR 235,919,585	EUR 0

aQIV, adjuvanted quadrivalent influenza vaccine; HD-QIV, high-dose quadrivalent influenza vaccine; QIVe, egg-based quadrivalent influenza vaccine. ^a^ In the current scenario of the model, persons were vaccinated according to age-based indication, i.e., the QIVe for those aged 50–59 years and the HD-QIV for those aged ≥60 years. In the alternative scenario, all persons aged ≥50 years were vaccinated with the aQIV. ^b^ Total for the current scenario includes both QIVe and HD-QIV costs.

**Table 4:** Comparative clinical outcomes and cost analyses.

Parameter		Current Scenario ^a^ (QIVe or HD-QIV)	Alternative Scenario ^a^ (aQIV)	Difference	Current Scenario ^a^ (QIVe or HD-QIV)	Alternative Scenario ^a^ (aQIV)	Difference
Clinical outcomes					QALY per vaccination		
	QALY loss	21,191	20,950	−241	0.00233	0.00230	−0.00003
	Life years lost	31,060	30,742	−318	0.00342	0.00338	−0.00003
Events					Events per vaccination		
	Symptomatic influenza cases	165,050	162,151	−2899	1.815%	1.783%	−0.00032
	Primary care visits	107,884	106,171	−1713	1.186%	1.167%	−0.00019
	ED visits	24,213	23,829	−384	0.266%	0.262%	−0.00004
	Hospitalizations	15,064	14,907	−157	0.166%	0.164%	−0.00002
	Deaths	3895	3860	−35	0.043%	0.042%	0.00000
Costs					Costs per vaccination		
	Primary care visits	EUR 7,358,761	EUR 7,241,929	EUR −116,832	EUR 0.8091	EUR 0.7963	EUR −0.013
	ED visits	EUR 6,222,855	EUR 6,124,057	EUR −98,798	EUR 0.6842	EUR 0.6734	EUR −0.011
	Hospitalizations	EUR 87,514,956	EUR 86,603,793	EUR −911,163	EUR 9.6225	EUR 9.5223	EUR −0.100
	Vaccination	EUR 439,617,397	EUR 354,152,222	EUR −85,465,175	EUR 48.3371	EUR 38.9400	EUR −9.397
	Total	EUR 540,713,969	EUR 454,122,002	EUR −86,591,968	EUR 59.4530	EUR 49.9319	EUR −9.521

aQIV, adjuvanted quadrivalent influenza vaccine; HD-QIV, high-dose quadrivalent vaccine; ED, emergency department; QALY, quality-adjusted life year; QIVe, egg-based quadrivalent vaccine. ^a^ In the current scenario of the model, persons were vaccinated according to age-based indication, i.e., the QIVe for those aged 50–59 years and the HD-QIV for those aged ≥60 years. In the alternative scenario, all persons aged ≥50 years were vaccinated with the aQIV.

## Data Availability

The original de-identified data used in this analysis were obtained from published sources. Interested researchers with reasonable and ethical inquiries may contact the authors for additional information.

## Supplementary Materials

The following supporting information can be downloaded at https://www.mdpi.com/article/10.3390/vaccines13030323/s1: Table S1: Influenza incidence, cases per 100,000 in Spain, 2013–2014; Table S2: Mortality; Table S3: Employment characteristics of the Spanish population, 2023; Table S4. Spanish population requiring care by a family member, 2008; Table S5: Tender price of the QIVe by region of Spain; Table S6: Summary of model scenarios used in the sensitivity analysis; Table S7: Summary of results from each model scenario in the sensitivity analysis.
