# Supplementary material for: Cost-Effectiveness of Adjuvanted Influenza Vaccine Compared with Standard and High-Dose Influenza Vaccines for Persons Aged ≥50 Years in Spain
Source: Vaccines (Basel). 2025 Mar 19;13(3):323. doi: 10.3390/vaccines13030323 (PMC11945739; doi:10.3390/vaccines13030323)
Supplement: Supplementary file 1 [file vaccines-13-00323-s001.zip › vaccines-3459963-supplementary.pdf]

Supplement to:

Cost-Effectiveness of Adjuvanted Influenza Vaccine Compared with Standard and High-Dose Influenza Vaccines for Persons Aged ≥50 Years in Spain

Alberto Perez-Rubio [1], Roberto Flores [2], Jesus Ruiz Aragon [3], Javier Sanchez [4], Sergio Marquez-Peláez [5], Piedad Alvarez [6], Andres Osorio Muriel [7] and Joaquin Mould-Quevedo [8,*]

[1] Hospital Clínico Universitario de Valladolid, 47003 Valladolid, Spain; albertoprz@gmail.com
[2] Medical Scientific Liaison, CSL Seqirus, 08027 Barcelona, Spain; roberto.flores@seqirus.com
[3] Hospital Universitario Puerta del Mar, 11009 Cádiz, Spain; reducido@hotmail.com
[4] Modeling and Simulation, Evidera, Torre Nozar, Titan, 15, 28045 Madrid, Spain
[5] Department of Economics, Economic Analysis, Faculty of Business Pablo de Olavide University, 41013 Seville, Spain; smarpel@upo.es
[6] Evidence, Modeling, and Synthesis, Evidera, 500 Totten Pond Road, Waltham, MA 02451, USA; piedad.alvarez@evidera.com
[7] Modeling and Simulation, Evidera, Bogota CO Calle 90, Bogota 110221, Colombia; andres.osoriomuriel@evidera.com
[8] Global Health Economics, CSL Seqirus, Summit, NJ 07901, USA
* Correspondence: Joaquin.Mould-Quevedo@seqirus.com

Table S1. Influenza incidence, cases per 100,000 in Spain, 2013–2020 [1-7].

Age group	Influenza season						
	**2013–2014**	**2014–2015**	**2015–2016**	**2016–2017**	**2017–2018**	**2018–2019**	**2019–2020**
0-4 years	3,571.99	4,496.66	5,886.82	3,361.12	5,110.00	4,857.60	6,244.70
5-14 years	2,198.69	4,960.07	4,362.50	2,827.39	4,242.00	3,710.90	4,995.60
15-64 years	1,350.59	1,975.02	1,538.37	1,442.20	1,971.00	1,435.70	1,640.20
≥65 years	532.94	1,103.97	623.29	942.10	1,163.00	667.50	545.40
Adjusted for age	1,450.14	2,366.93	2,004.16	1,649.96	2,313.73	1,801.10	2,160.80

Table S2. Absolute influenza-related deaths [8]

Age group	2013	2014	2015	2016	2017	2018	2019
0-50 years	221	221	221	221	221	221	221
50-59 years	280	280	280	280	280	280	280
60-64 years	194	194	194	194	194	194	194
65-74 years	558	558	558	558	558	558	558
≥75 years	3602	3602	3602	3602	3602	3602	3602
Total	4854	4854	4854	4854	4854	4854	4854

Total data from Leon Gomez (average 2006-2012) [8], adjusted by age based on the average observed proportions in 2013-2019 from the INE.

Table S3. Employment characteristics of the Spanish population, 2023 [9,10].

Age group	Number of Spanish residents	Proportion of Spanish population, %	Employed, n (%)	Labor cost per hour[a], €
0-50 years	20,909,620	50.4	13,753,300 (65.8)	13.32
50-59 years	7,475,397	18.0	5,353,600 (71.6)	15.86
60-64 years	3,214,012	7.8	1,572,900 (48.9)	16.22
65-74 years	2,702,822	6.5	270,300 (10.0)	16.22
≥75 years	7,162,119	17.3	55,400 (0.8)	16.22
Total	41,463,970	100	21,005,500 (50.7)	

[a]Assumes an 8-hour day for all employed persons.

Table S4. Spanish population requiring care by a family member, 2008 [11].

Age group	Number of Spanish residents	Proportion of Spanish population, %	Cared for by a family member, n (%)
65-74 years	4,974,175	18.4	916,665 (50.4)
≥75 years	4,890,766	45.1	2,207,740 (49.6)
Total	9,864,941	31.7	3,124,405

Table S5. Tender prices of QIVe by region of Spain.

Company	Vaccine	Technology	Campaign	Region	QTy Doses	Price Doses (unit)	Total (Precio x Dosis)	Reference
Sanofi	Vaxigrip Tetra	QIV	23/24	Andalucía	1,300,000	5.20 €	6,760,000.00 €	[12]
Mylan	Influvac Tetra	QIV	23/24	Aragón	313,569	4.09 €	1,282,497.21 €	[13]
Sanofi	Vaxigrip Tetra	QIV	23/24	Asturias	7,000	5.49 €	38,430.00 €	[14]
Sanofi	Vaxigrip Tetra	QIV	23/24	Baleares	100,000	8.79 €	879,000.00 €	[15,16]
Sanofi	Vaxigrip Tetra	QIV	23/24	Canarias	100,000	3.90 €	390,000.00 €	[17]
Sanofi	Vaxigrip Tetra	QIV	23/24	Cantabria	115,000	4.30 €	494,500.00 €	[18]
Sanofi	Vaxigrip Tetra	QIV	23/24	Castilla la Mancha	100,000	5.00 €	500,000.00 €	[19]
Sanofi	Vaxigrip Tetra	QIV	23/24	Castilla y León	300,000	4.89 €	1,467,000.00 €	[20]
Sanofi	Vaxigrip Tetra	QIV	23/24	Catalunya	326,700	4.90 €	1,600,830.00 €	[21]
Mylan	Influvac Tetra	QIV	23/24	Catalunya	302,500	4.55 €	1,376,375.00 €	[21]
Mylan	Influvac Tetra	QIV	23/24	Catalunya	355,300	4.40 €	1,563,320.00 €	[21]
Mylan	Influvac Tetra	QIV	23/24	Catalunya	280,500	4.60 €	1,290,300.00 €	[21]
Sanofi	Vaxigrip Tetra	QIV	23/24	Ceuta	3,000	5.51 €	16,530.00 €	[22]
Mylan	Influvac Tetra	QIV	23/24	Comunidad de Madrid	750,000	3.35 €	2,512,500.00 €	[23]
Sanofi	Vaxigrip Tetra	QIV	23/24	Comunidad Foral de Navarra	175,000	4.00 €	700,000.00 €	[24]
Mylan	Influvac Tetra	QIV	23/24	Comunidad Valenciana	65,000	4.09 €	265,850.00 €	[25]
Sanofi	Vaxigrip Tetra	QIV	23/24	Extremadura	260,000	4.70 €	1,222,000.00 €	[26]
Mylan	Influvac Tetra	QIV	23/24	Galicia	670,000	4.15 €	2,780,500.00 €	[27]
Sanofi	Vaxigrip Tetra	QIV	23/24	Melilla	14,000	9.40 €	131,600.00 €	[28]
GSK	Fluarix Tetra	QIV	23/24	País Vasco	620,000	7.50 €	4,650,000.00 €	[29]
Mylan	Influvac Tetra	QIV	23/24	Región de Murcia	302,000	3.35 €	1,011,700.00 €	[30]
Sanofi	Vaxigrip Tetra	QIV	23/24	Rioja	37,000	4.30 €	159,100.00 €	[31]
Mylan	Influvac Tetra	QIV	23/24	Rioja	11,800	3.80 €	44,840.00 €	[31]

Company	Vaccine	Technology	Campaign	Region	QTy Doses	Price Doses (unit)	Total (Precio x Dosis)	Reference
**Total**					6,508,369.00 €	114.26 €	31,136,872.21 €	
**Weighted average**							4.78 €	

Table S6. Summary of model scenarios used in the sensitivity analysis.

Parameter	Age group	Base case	Scenario	Scenario number	Reference
		Healthcare system	Public	1	
Cost and benefit discount rates		3%	0%	2	
			1.5%	3	
Influenza vaccine coverage, high risk	0–50 years	0%	0%	4	[32-35]
	50-59 years	24.54%	24.54%		
	60-64 years	36.72%	24.54%		
	65-74 years	61.57%	54.70%		
	≥75 years	75.27%	63.85%		
Influenza vaccine coverage, low risk	0–50 years	0%	0%		
	50-59 years	0%	0%		
	60-64 years	0%	0%		
	65-74 years	0%	0%		
	≥75 years	0%	0%		
rVE		aQIV vs HD-QIV: 1.4%	Use of values from Domnich 2022 by age group:<br>60–64 years: 7.8%<br>65–74 years: 7.8%<br>≥75 years: 12.5%	6a	[36]
		aQIV vs QIVe: 20%	aQIV vs QIVe: 13.9%	6b	[37,38]
			aQIV vs QIVe: 34.6%	6c	[37,39]
Cost by influenza vaccine type		aQIV: 13.00 €<br>QIVe: 4.78 €<br>HD-QIV: 25 €	QIVe: 3.35 €[a]	7	[40]
			QIVe: 9.4 €[b]	8	[40]

aQIV, adjuvanted quadrivalent influenza vaccine; HD-QIV, high-dose quadrivalent influenza vaccine; INE, Instituto Nacional de Estadistica (National Institute of Statistics); QIVe, egg-based quadrivalent influenza vaccine; rVE, relative vaccine effectiveness.

[a]Cost as listed in Murcia/Madrid.

[b]Cost as listed in Melilla.

Table S7. Summary of results from each model scenario in the sensitivity analysis.

	Scenario	Incremental cost	QALY gained	Increase in years of life gained	ICER	Cost per QALY gained	Cost per year of life gained
1	Public health system perspective	-93,324,861.46 €	241.02	318.04	Intervention dominates	-387,202.67 €	-293,441.63 €
2	Discount rate 0%	-86,591,967.67 €	301.26	401.43	Intervention dominates	-287,430.76 €	-215,710.71 €
3	Discount rate 1.5%	-86,591,967.67 €	267.49	354.59	Intervention dominates	-323,716.51 €	-244,202.43 €
4	Influenza vaccine coverage per Garcia 2016 [35]	-70,929,550.96 €	220.08	285.49	Intervention dominates	-322,289.53 €	-248,451.35 €
6a	rVE, aQIV vs HD-QIV:<br>60–64 years: 7.8%<br>65–74 years: 7.8%<br>≥75 years: 12.5%	-91,230,973.19 €	1245.10	1971.34	Intervention dominates	-73,271.98 €	-46,278.72 €
6b	rVE, aQIV vs QIVe: 13.9%	-86,480,213.18 €	215.98	299.06	Intervention dominates	-400,407.72 €	-289,176.08 €
6c	rVE, aQIV vs QIVe: 34.6%	-86,859,445.64 €	300.96	363.46	Intervention dominates	-288,605.98 €	-238,980.14 €
7	QIVe, 3.35 € (Murcia/Madrid)	-84,917,789.57 €	241.02	318.04	Intervention dominates	-352,321.92 €	-267,007.25 €
8	QIVe, 9.4 € (Melilla)	-92,000,850.78 €	241.02	318.04	Intervention dominates	-381,709.38 €	-289,278.54 €

aQIV, adjuvanted quadrivalent influenza vaccine; HD-QIV, high-dose quadrivalent influenza vaccine; ICER, incremental cost-effectiveness analysis; QALY, quality-adjusted life years gained; QIVe, egg-based quadrivalent influenza vaccine; rVE, relative vaccine effectiveness.

References

1. Instituto de Salud Carlos III. Informe de vigilancia de la gripe en España. Temporada 2019-2020 (desde la semana 40/2019 hasta la semana 20/2020). Available online: https://vgripe.isciii.es/documentos/20192020/InformesAnuales/Informe_Vigilancia_GRIPE_2019-2020_03092020.pdf (accessed on 4 October 2024).
2. Instituto de Salud Carlos III. Informe de vigilancia de la gripe en España. Temporada 2018-2019 (desde la semana 40/2018 hasta la semana 20/2019). Available online: https://vgripe.isciii.es/documentos/20182019/InformesAnuales/Informe_Vigilancia_GRIPE_2018-2019_22julio2019.pdf (accessed on 4 October 2024).
3. Instituto de Salud Carlos III. Informe de vigilancia de la gripe en España. Temporada 2016-2017 (desde la semana 40/2016 hasta la semana 20/2017). Available online: https://cne.isciii.es/documents/d/cne/vigilancia-de-la-gripe-en-espana-informe-temporada-2016-2017 (accessed on 4 October 2024).
4. Instituto de Salud Carlos III. Informe de vigilancia de la gripe en España. Temporada 2017-2018 (desde la semana 40/2017 hasta la semana 20/2018). Available online: https://cne.isciii.es/documents/d/cne/vigilancia-de-la-gripe-en-espana-informe-temporada-2017-2018 (accessed on 4 October 2024).
5. Instituto de Salud Carlos III. Informe de vigilancia de la gripe en España. Temporada 2015-2016 (desde la semana 40/2015 hasta la semana 20/2016). Available online: https://cne.isciii.es/documents/d/cne/vigilancia-de-la-gripe-en-espana-informe-temporada-2015-2016-pdf (accessed on 4 October 2024).
6. Instituto de Salud Carlos III. Informe de vigilancia de la gripe en España. Temporada 2014-2015 (desde la semana 40/2014 hasta la semana 20/2015). Available online: https://cne.isciii.es/documents/d/cne/vigilancia-de-la-gripe-en-espana-informe-temporada-2014-2015-pdf (accessed on 4 October 2024).
7. Delgado-Sanz, C.; Jiménez-Jorge, S.; Pozo, F.; Gómez-Barroso, D.; León-Gómez, I.; de Mateo, S.; Larrauri, A. Vigilancia de la gripe en España. Temporada 2013-2014 (desde la semana 40/2013 hasta la semana 20/2014). *Boletín epidemiológico Semanal* **2014**, *22*, 146-166.
8. León-Gómez, I.; Delgado-Sanz, C.; Jiménez-Jorge, S.; Flores, V.; Simón, F.; Gómez-Barroso, D.; Larrauri, A.; de Mateo Ontañón, S. [Excess mortality associated with influenza in Spain in winter 2012]. *Gac Sanit* **2015**, *29*, 258-265, doi:10.1016/j.gaceta.2015.01.011.
9. Instituto Nacional de Estadistica. Resultado por Comunidades autónomas (desde el trimestre 1/2008). Componentes del coste laboral total. Available online: https://www.ine.es/jaxiT3/Tabla.htm?t=6062 (accessed on 12 November 2024).
10. Instituto Nacional de Estadistica. Ocupados por sexo y grupo de edad. Valores absolutos y porcentajes respecto del total de cada sexo. Available online: https://www.ine.es/jaxiT3/Tabla.htm?t=4076&L=0 (accessed on 9 October 2024).
11. Instituto Nacional de Estadistica. Encuesta de discapacidad, autonomía personal y situaciones de dependencia 2008: discapacidades, deficiencias y estado de salud. Resultados nacionales: cifras relativas. Tasa de población con alguna discapacidad o limitación por edad y sexo. Available online: https://www.ine.es/jaxi/Tabla.htm?path=/t15/p418/a2008/hogares/p01/modulo1/l0/&file=02001.px&L=0 (accessed on 9 October 2024).
12. Junta de Andalucía. CCA:+6.+PJ7JD7 Acuerdo marco con una única empresa por lote, por el que se fijan las condiciones para el suministro de tracto sucesivo y precio unitario de vacuna antigripal destinada al Programa de Vacunaciones de Andalucía,

campaña 2023-2024. Available online: https://www.juntadeandalucia.es/haciendayadministracionpublica/apl/pdc_sirec/perfiles-licitaciones/detalle-licitacion.jsf?idExpediente=531651 (accessed on 4 December 2024).
13. Gobierno de España. Plataforma de Contratacion del Sector Publico: Expediente: SAS-SGE-2023-42. Available online: https://contrataciondelestado.es/wps/poc?uri=deeplink:detalle_licitacion&idEvl=st4PYafAE6geC9GJQOEBkQ%3D%3D (accessed on 2 December 2024).
14. Gobierno del Principado de Asturias. Documentos electrónicos emitidos por el Principado de Asturias, número de referencia 14612415324201513343 [login required]. Available online: https://www62.asturias.es/ (accessed on 15 January 2025).
15. Gobierno de España. Plataforma de Contratacion del Sector Publico: Expediente: PRO11 2023 12880. Available online: https://contrataciondelestado.es/wps/poc?uri=deeplink%3Adetalle_licitacion&idEvl=OBymDxcgIdKopEMYCmrbmw%3D%3D (accessed on 2 December 2024).
16. Gobierno de España. Plataforma de Contratacion del Sector Publico: Expediente: 2021/ETSAE0287/00000463E. Available online: https://contrataciondelestado.es/wps/poc?uri=deeplink%3Adetalle_licitacion&idEvl=i%2Bv38mJHKLnnSoTX3z%2F7wA%3D%3D (accessed on 4 December 2024).
17. Gobierno de España. Plataforma de Contratacion del Sector Publico: Expediente: 02/CONS/DGSP/2023. Available online: https://contrataciondelestado.es/wps/poc?uri=deeplink%3Adetalle_licitacion&idEvl=Uh9akaL3pAY%2Bk2oCbDosIw%3D%3D (accessed on 4 December 2024).
18. Gobierno de España. Plataforma de Contratacion del Sector Publico: Expediente: 10.2.10/23. Available online: https://contrataciondelestado.es/wps/poc?uri=deeplink%3Adetalle_licitacion&idEvl=%2FWwhozokA5q5HQrHoP3G5A%3D%3D (accessed on 4 December 2024).
19. Gobierno de España. Plataforma de Contratacion del Sector Publico: Expediente: 2023/007980. Available online: https://contrataciondelestado.es/wps/poc?uri=deeplink%3Adetalle_licitacion&idEvl=Iu2C4QH969fyoM4us5k4vw%3D%3D (accessed on 4 December 2024).
20. Gobierno de España. Plataforma de Contratacion del Sector Publico: Expediente: 182/2023. Available online: https://contrataciondelestado.es/wps/poc?uri=deeplink%3Adetalle_licitacion&idEvl=pmcEYn4kqsVPpzdqOdhuWg%3D%3D (accessed on 4 December 2024).
21. Catalunya, G.d. Plataforma de Serveis de Contractació Pública: Adquisició de vacunes per a l'any 2023 (SA-2023-39). Available online: https://contractaciopublica.cat/es/detall-publicacio/200156331 (accessed on 4 December 2024).
22. Gobierno de España. Plataforma de Contratacion del Sector Publico: Expediente: 19251/23. Available online: https://contrataciondelestado.es/wps/poc?uri=deeplink%3Adetalle_licitacion&idEvl=EjOGS%2BYgL0rzAq95uGTrDQ%3D%3D (accessed on 4 December 2024).
23. Comunidad de Madrid. Contratos basados en el acuerdo marco 202101AM0001, relativo al suministro de vacunas frente a la gripe estacional, 2 lotes (lote 1 y lote 3) para la campaña de vacunación antigripal de la temporada 2023-2024 para la Comunidad de Madrid. Available online: https://contratos-publicos.comunidad.madrid/contrato-publico/contratos-basados-acuerdo-marco-202101am0001-relativo-suministro-vacunas-frente (accessed on 4 December 2024).

24. Gobierno de Navarra. Digital budget records [restricted access]. Available online: https://administracionelectronica.navarra.es/validarCSV/default.aspx (accessed on 4 December 2024).
25. Gobierno de España. Plataforma de Contratacion del Sector Publico: Expediente: 725/2021 Tercer Basado Lote1. Available online: https://contrataciondelestado.es/wps/poc?uri=deeplink%3Adetalle_licitacion&idEvl=LDBtSC1j%2B%2BHua%2Fi14w%2FPLA%3D%3D (accessed on 4 December 2024).
26. Gobierno de España. Plataforma de Contratacion del Sector Publico: Expediente: CS/99/1123044902/23/PNSP. Available online: https://contrataciondelestado.es/wps/poc?uri=deeplink%3Adetalle_licitacion&idEvl=HopD%2BsAA%2FDk4NavIWzMcHA%3D%3D (accessed on 4 December 2024).
27. Xunta de Galicia; Consellería de Sanidade. Datos xerais. Available online: https://www.contratosdegalicia.gal/licitacion?OP=50&N=819632&lang=gl (accessed on 4 December 2024).
28. Gobierno de España. Plataforma de Contratacion del Sector Publico: Expediente: 181/2021/CMA. Available online: https://contrataciondelestado.es/wps/poc?uri=deeplink%3Adetalle_licitacion&idEvl=Q0aw7etnXBGmq21uxhbaVQ%3D%3D (accessed on 4 December 2024).
29. Gobierno Vasco. Suministro de vacunas frente a la gripe estacional - Perfil de contratante. Available online: https://www.contratacion.euskadi.eus/webkpe00-kpeperfi/es/contenidos/anuncio_contratacion/exposakisap2021000431/es_doc/index.html?ruta=informacionAmpliadaAnuncios&busquedaAvanzada (accessed on 4 December 2024).
30. Gobierno de España. Plataforma de Contratacion del Sector Publico: Expediente: CS/9999/1101105289/23/PNSP. Available online: https://contrataciondelestado.es/wps/poc?uri=deeplink%3Adetalle_licitacion&idEvl=kHqM2iNSHV2ExvMJXBMHHQ%3D%3D (accessed on 4 December 2024).
31. Gobierno de La Rioja. Consulta expedientes: Expediente nº.06-3-7.07-0019/2023 - Adjudicación. Available online: https://www.larioja.org/contratacion-publica/es/licitaciones/consulta-expedientes?homepage=%3Fp_colateral%3D06-3-7.07-0019%2F2023%26p_dia_ini%3D28%26p_mes_ini%3D07%26p_ano_ini%3D2023%26p_dia_fin%3D28%26p_mes_fin%3D07%26p_ano_fin%3D2023%26p_todas%3DN (accessed on 4 December 2024).
32. Instituto Nacional de Estadistica. Población residente por fecha, sexo y edad. Available online: https://www.ine.es/jaxiT3/Tabla.htm?t=59583&L=0 (accessed on 9 October 2024).
33. Ministerio de Sanidad. Sistema de Información de Vacunaciones (SIVAMIN). Available online: https://pestadistico.inteligenciadegestion.sanidad.gob.es/publicoSNS/S/sivamin (accessed on 21 November 2024).
34. Junta de Castilla de León. Seguimiento de las coberturas de la campaña de vacunación gripe y COVID-19 2023-2024. Available online: https://www.saludcastillayleon.es/profesionales/en/vacunaciones/campana-vacunacion-frente-gripe-covid-19-temporada-2023-202/seguimiento-coberturas-campana-vacunacion-gripe-covid-19-20 (accessed on 21 November 2024).
35. García, A.; Ortiz de Lejarazu, R.; Reina, J.; Callejo, D.; Cuervo, J.; Morano Larragueta, R. Cost-effectiveness analysis of quadrivalent influenza vaccine in Spain.

Human vaccines & immunotherapeutics **2016**, *12*, 2269-2277, doi:10.1080/21645515.2016.1182275.
36. Domnich, A.; de Waure, C. Comparative effectiveness of adjuvanted versus high-dose seasonal influenza vaccines for older adults: a systematic review and meta-analysis. *Int J Infect Dis* **2022**, *122*, 855-863, doi:10.1016/j.ijid.2022.07.048.
37. McConeghy, K.W.; Davidson, H.E.; Canaday, D.H.; Han, L.; Saade, E.; Mor, V.; Gravenstein, S. Cluster-randomized Trial of Adjuvanted Versus Nonadjuvanted Trivalent Influenza Vaccine in 823 US Nursing Homes. *Clinical Infectious Diseases* **2021**, *73*, e4237-e4243, doi:10.1093/cid/ciaa1233.
38. Coleman, B.L.; Sanderson, R.; Haag, M.D.M.; McGovern, I. Effectiveness of the MF59-adjuvanted trivalent or quadrivalent seasonal influenza vaccine among adults 65 years of age or older, a systematic review and meta-analysis. *Influenza Other Respir Viruses* **2021**, *15*, 813-823, doi:10.1111/irv.12871.
39. Calabrò, G.E.; Boccalini, S.; Bonanni, P.; Bechini, A.; Panatto, D.; Lai, P.L.; Amicizia, D.; Rizzo, C.; Ajelli, M.; Trentini, F.; et al. Valutazione di Health Technology Assessment (HTA) del vaccino antinfluenzale quadrivalente adiuvato: Fluad Tetra. Available online: https://www.ijph.it/hta-vaccino-antinfluenzale-quadrivalente-adiuvato-fluad-tetra (accessed on 23 May 2023).
40. Ministerio de Sanidad Consumo y Bienestar Social. Acuerdo Marco para la Seleccion de Suministradores de Vacunas Frente a la Gripe Estacional (INGESA) y Ciudades de Ceuta y Melilla y Varias Comunidades Autonomas. Available online: https://contrataciondelestado.es/wps/wcm/connect/7c41cd41-00c8-4c07-be3d-272d29585268/DOC20210419131140PCAP+Gripe+2021-2025.pdf?MOD=AJPERES (accessed on 9 October 2024).
